# Fused Deposition Modeling of Chemically Resistant Microfluidic Chips in Polyvinylidene Fluoride

**DOI:** 10.3390/mi15111391

**Published:** 2024-11-17

**Authors:** Christof Rein, Leonhard Hambitzer, Zahra Soraya, Han Zhang, Henning J. Jessen, Frederik Kotz-Helmer, Bastian E. Rapp

**Affiliations:** 1 Laboratory of Process Engineering, NeptunLab, Department of Microsystems Engineering (IMTEK), University of Freiburg, 79110 Freiburg im Breisgau, Germany; christof.rein@neptunlab.org (C.R.); leonhard.hambitzer@neptunlab.org (L.H.); zahra.soraya@neptunlab.org (Z.S.); bastian.rapp@neptunlab.org (B.E.R.); 2 Institute of Organic Chemistry, Faculty of Chemistry and Pharmacy, University of Freiburg, 79104 Freiburg im Breisgau, Germany; han.zhang@ocbc.uni-freiburg.de (H.Z.); henning.jessen@oc.uni-freiburg.de (H.J.J.); 3 Centre of Integrative Biological Signaling Studies (CIBSS), University of Freiburg, 79104 Freiburg im Breisgau, Germany; 4 Freiburg Materials Research Center (FMF), University of Freiburg, 79104 Freiburg im Breisgau, Germany; 5 FIT Freiburg Center of Interactive Materials and Bioinspired Technologies, University of Freiburg, 79110 Freiburg im Breisgau, Germany

**Keywords:** 3D printing, additive manufacturing, fused deposition modeling, microfluidics, polyvinylidene fluoride, lab-on-a-chip

## Abstract

Fused deposition modeling (FDM) is well suited for microfluidic prototyping due to its low investment cost and a wide range of accessible materials. Nevertheless, most commercial FDM materials exhibit low chemical and thermal stability. This reduces the scope of applications and limits their use in research and development, especially for on-chip chemical synthesis. In this paper, we present FDM fabrication of microfluidic chips with polyvinylidene fluoride (PVDF) for applications that require high thermal or chemical resistance. Embedded microchannels with a minimum channel width and heights of ~200 µm × 200 µm were fabricated, and the resistance against common solvents was analyzed. A procedure was developed to increase the optical transmission to result in translucent components by printing on glass. Chips for fluid mixing were printed, as well as microreactors that were packed with a catalytically active phase and used for acetal deprotection with a conversion of more than 99%. By expanding the use of fluorinated polymers to FDM printing, previously challenging microfluidic applications will be conducted with ease at the lab scale.

## 1. Introduction

The field of microfluidics offers great potential to miniaturize processes in chemistry, biochemistry, and biology. Miniaturization of reactions and analytical processes can lead to a reduction in reaction time and reactant’s volume and facilitate parallelization [1,2]. The processing time can be reduced as mixing and heating occur faster within smaller volumes [3]. Furthermore, it is advantageous for analytical applications to run the experiment within a smaller sample size. This especially holds true for biological or medical analytics, where the samples are difficult to acquire or the sample volume is very small. Furthermore, for chemical applications, hazardous reactions can be scaled down in order to reduce the risk of critical incidents [4,5].

Over the past few decades, additive manufacturing (AM) has been proven as a convenient tool for microfluidics fabrication due to its low cost [6], arbitrary design [7], and one-step fabrication [8]. Assisted by computer-aided design (CAD), the 3D printing process allows for facile alternation and adaptation of prospective geometries at various stages of the development process, decreasing the concept-to-chip time [9]. Different technologies of AM have been successfully used for microfluidic fabrication such as digital light processing (DLP) [10,11], laser-based stereolithography (SL) [12], two-photon lithography [13,14], inkjet printing [15], suspended liquid subtractive lithography (SLSL) [16], and fused deposition modeling (FDM) [17,18,19]. The scope of microfluidic devices fabricated by AM is expanded continuously, and AM-fabricated microfluidics have been used in cell culturing to create microgels for cell encapsulation [20] and encapsulations for stem cell engineering [21,22]. Three-dimensionally printed integrated microfluidics have been utilized for flow rate sensing [23] and amperometric detection in electrophoresis [24]. Furthermore, on-chip chemical synthesis has been shown to synthesize PET tracers [25], nucleoside analogs [26], and self-heating microreactors have been demonstrated [27]. FDM printing stands out as one of the most widespread AM technologies due to its simplicity and low cost of investment and operation. A wide variety of thermoplastic materials can be processed with FDM, such as polylactic acid (PLA), acrylonitrile butadiene styrene (ABS), poly(ethylene terephthalate) (PET), and polycarbonate (PC). Recently, our lab also demonstrated FDM fabrication of microfluidic chips in poly(methyl methacrylate) (PMMA) and polystyrene (PS) [17,18]. These materials are used for large-scale production methods like hot embossing or injection molding. This makes FDM printing a preferable AM method for prototyping, as the transfer from lab-scale to industrial processes is easier to conduct [28].

Despite their industrial abundance and good optical properties, most FDM materials suffer from low chemical resistance, especially against organic solvents [29,30]. Furthermore, most of these materials have low heat resistance, and their Vicat softening points are commonly located well below 100 °C [31]. This limits the versatility of the devices manufactured with FDM, as they prevent the application of microfluidic chips in environments that call for high-performance material properties. A potential material of choice for this could be fused silica, which has outstanding chemical and heat resistance. FDM printing of silica by using a thermoplastic nanocomposite, which is subsequently debinded and sintered, has been demonstrated [32]. Nevertheless, the process is challenging and requires a multi-step protocol in order to generate functional chips. Therefore, there is a demand for material that can be processed with FDM and can yield microfluidic chips with improved thermal and chemical stability compared to conventional FDM polymers.

Fluorinated polymers are superior regarding their chemical resistance, good mechanical strength, and UV stability. Additionally, they mostly exhibit high melting points [33]. The reason for their low reactivity and swelling in organic solvents is the short proximity within the carbon–fluorine bond [34]. The distance between both atoms is 1.35 Å, making it the shortest of all covalent bonds in organic molecules and the reason why intermolecular interactions with the carbon atoms of the polymer backbone are reduced in comparison to non-fluorinated polymers [35]. Additional to the chemical resistance, their thermal stability can be advantageous for microfluidic reactions that require high temperatures to accelerate reaction speed. For example, a typical polymerase chain reaction protocol involves heating to >90 °C, which exceeds the working temperature of PLA [36]. In comparison, fluorinated thermoplasts are well suited for these temperatures due to their typically high melting points of common commercial fluorinated thermoplasts such as fluorinated ethylene propylene (FEP, m_p_: 260 °C) or polytetrafluoroethylene (PTFE, m_p_: 327 °C) [37]. Nevertheless, these temperatures are not suitable for most FDM printers. Polyvinylidene fluoride (PVDF, m_p_: 177 °C) is a thermoplastic polymer that exhibits the advantages of fluorinated polymers and has a melting point that is low enough to be processed with generic FDM printers [38]. The surface energy of the difluoromethylene group (-CF_2_-) is one of the lowest found in organic molecules at ~18 mN/m [39]. Its maximum continuous service temperature is 150 °C and therefore higher than most conventional FDM materials [31]. These outstanding properties make fluorinated polymers the ideal material for microfluidic chemical reactors.

In this work, we demonstrate for the first time FDM printing of microfluidic chips in PVDF in order to fabricate high-performance microfluidics in a simple one-step protocol. We show how to eliminate warping of the material during printing and to increase transparency by printing directly on a glass substrate. Furthermore, we demonstrate the high chemical resistance of the functional microfluidic chips made of PVDF in applications that would be unfeasible with conventional FDM materials. Applying our FDM protocol to prototype microfluidics with high-performance properties will open new possibilities for lab-on-a-chip (LoC) applications in the field of chemical synthesis.

## 2. Materials and Methods

### 2.1. Materials

The filament 3DXTech FluorX PVDF with a diameter of 2.85 mm was purchased from Filamentworld (Neu-Ulm, Germany). Acetone, acetone-d_6_, 2-propanol, methanol (MeOH), dichloromethane (DCM), dimethylformamide (DMF), tetrahydrofuran (THF), toluene, *n*-heptane, diphenyl(2,4,6-trimethylbenzoyl) phosphine oxide (TPO), and strongly acidic ion exchanger resin Amberlyst 15 (hydrogen form, dry) were purchased from Merck (Darmstadt, Germany). Bromothymol blue, phenolphthalein, and toluene sulfonic acid monohydrate were purchased from Carl Roth GmbH & Co., KG (Karlsruhe, Germany). Fluorolink MD700 (MD700) was obtained from Acota (Oswestry, UK). Elastosil RT 601 A/B was purchased from Wacker (Munich, Germany). Benzaldehyde dimethyl acetal was purchased from BLD Pharm (Shanghai, China).

### 2.2. Fused Deposition Modeling

The CAD models for the FDM printing were designed using Autodesk Inventor Professional 2023 (Autodesk, San Rafael, CA, USA) and exported as STL files. The STL files were sliced using the slicing software Ultimaker Cura 4.7.1. (Ultimaker B.V., Utrecht, The Netherlands). Three-dimensional printing was conducted on a commercial 3D printer (Ultimaker 3, Ultimaker B.V., Utrecht, The Netherlands). A nozzle diameter of 0.4 mm was chosen. The printing temperature of the initial layer was 190 °C and the printing temperature of the residual layers was 230 °C. The built plate temperature was set to 95 °C for all layers. The printing parameters were optimized for the fabrication of microfluidic devices. The layer thickness was set to 100 µm and the line width to 350 µm. The printing and infill speeds were set to 30 mm/s, and the infill density and wall infill flowrate were set to 100% and 87%, respectively. To ensure the adhesion of the prints to the printing bed, adhesion spray 3DLac (niceshops GmbH, Germany) was applied to the platform, and a brim of 7 mm width was added while printing the first layer.

For evaluation of the channel resolution, a series of embedded square-shaped channels with different diameters were printed, cut with a precision saw, Diadisc 5200 (Mutronic, Rieden am Forggensee, Germany), and then polished with a diamond mill. The cross-section was analyzed by a light microscope of type VHX6000 (Keyence, Osaka, Japan).

### 2.3. Casting of PDMS and PFPE Samples

Two reference materials were cast to compare the swelling in common solvents with the 3D-printed PVDF. Polydimethylsiloxane (PDMS) samples were cast by mixing Elastosil RT 601 A/B in a ratio of 9:1 (A to B, by weight) and poured into 200 µL cavities. Prior to casting, entrapped air bubbles were removed using a vacuum desiccator. The PDMS was cured at 65 °C for 2 h, then allowed to cool to room temperature and carefully removed from the mold. For the second benchmark substance, the photoinitiator TPO (1 wt%) was dissolved in the highly fluorinated perfluoropolyether (PFPE) methacrylate MD700 at room temperature. The photoresist was cast into 200 µL cavities and cured at 365 nm for 10 min. After curing, the photo-crosslinked PFPE acrylates were carefully removed from the mold. A comparable mixture has been utilized by our group for 3D printing of microfluidic chips via vat-photopolymerization [39].

### 2.4. Solvent Compatibility

Solvent compatibility of the printed PVDF material was determined using 10 mm × 10 mm × 3 mm printed blocks of PVDF. The weight of each block was determined three times, and the block was immersed in 20 mL of the respective solvent (water, MeOH, DCM, DMF, THF, toluene, acetone, and *n*-heptane) for 24 h. After immersion, the blocks were cleaned with a tissue, and the weight after swelling was determined immediately. The block was then re-immersed in the same solvent and, after weighting, the next eight blocks. A total of three measurements were taken for each of the three blocks.

### 2.5. White Light Interferometry and Roughness Determination

The surface roughness of 3D-printed samples was analyzed by white light interferometry (WLI) (NewView 9000, Zygo, Middlefield, CT, USA). The surface roughness measurements were carried out on an area of 1.6 × 1.6 mm^2^ at three different positions, calculating the surface roughness S_q_ and mean line roughness R_a_ with Gwyddion.

### 2.6. Scanning Electron Microscopy

Scanning electron microscopy (SEM) was carried out on a Quanta 250 FEG (FEI Inc., Valley City, ND, USA) with an accelerating potential of 5 kV. Samples were prepared with conductive tape on SEM sample holders and conductive silver paint and were sputtered with a gold–palladium layer of about 25 nm thickness prior to SEM imaging.

### 2.7. Microfluidic Experiments

To investigate the functionality of the FDM-printed microfluidic chips, the channels were filled with fluids using a syringe pump (Legato 210, KDScientific, Holliston, MA, USA). To connect the syringe with the chips, PTFE tubes were plugged onto a 3D-printed chip-to-world interface and fixed with epoxy glue. Pumping rates up to 10 mL/min were used to perfuse the microfluidic chips.

### 2.8. ^1^H-NMR Spectroscopy

The ^1^H-NMR spectra were measured on a Bruker Avance III HD 300 MHz NMR spectrometer. The solvent used was aceton-d_6_, which was also used as the reaction solvent. All signals were referenced to an internal solvent signal (2.05 ppm). The evaluation of NMR spectra was performed using the software MestreNova 15.0 (Mestrelab Research, Santiago de Compostela, Spain).

## 3. Results and Discussion

### 3.1. Optimization of Printing Parameters and Improvement of Transparency

**Figure 1:**
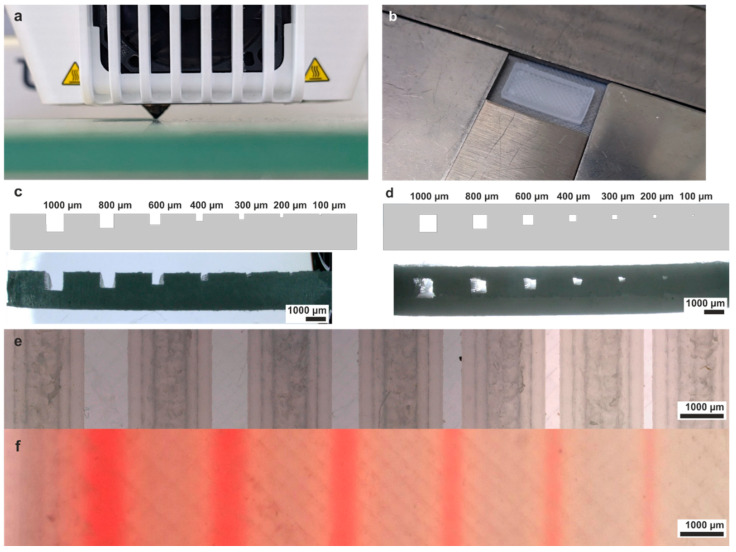
Fused deposition modeling of PVDF and optimization of the printing parameters. (**a**) The second extruder nozzle, which was the default setup for the Ultimaker 3, was removed in order to place a metal frame around the printed part. (**b**) The metal frame presses onto the brim and decreases the chance of warping during the printing. (**c**) CAD file and 3D-printed part of the calibration structure used for optimizing the wall infill flowrate for open 3D-printed channel structures. Open channels as small as 200 µm × 200 µm were successfully fabricated. (**d**) CAD file and 3D-printed structure of the calibration structure used for optimizing the wall infill flowrate for embedded 3D-printed channel structures. Embedded channels down to 200 µm × 200 µm were successfully fabricated. (**e**) Top view on open channel structures demonstrating that structures down to 200 µm × 200 µm were successfully fabricated. (**f**) Top view on embedded channel structures filled with dyed water. All channels down to 200 µm × 200 µm were functional and successfully filled.

A significant challenge when processing PVDF with an FDM printer is the poor adhesion of the printed structures to the built plate. This effect is called “warping” and can be attributed to the shrinkage that the polymer undergoes during the cooling after the melt extrusion. The adhesion is usually lower when printing structures with a spacious footprint, which is a challenge in the fabrication of microfluidic devices. Numerous adaptations to the printing process were made to reduce the warping of the printed devices. The build plate was heated to 95 °C to reduce the temperature gradient between the nozzle at 190 °C and the build plate. Furthermore, a brim adhesion geometry consisting of nine lines was generated within the slicing software around the footstep of the chips to increase the adhesion to the built plate. The width of the brim was chosen to be 7 mm. Nevertheless, many structures warped when fabricated with a brim. Therefore, the adhesion to the built plate was further increased by applying a commercial adhesion spray (3DLac, niceshops GmbH, Germany) before printing. Structures that were still warped were stabilized by placing steel plates on top of the brim (Figure 1b). As the printer is equipped with two print heads, the second nozzle was removed to avoid collision with the steel plates (Figure 1a). All structures were printed with 100% infill of the bulk phase.

**Figure 2:**
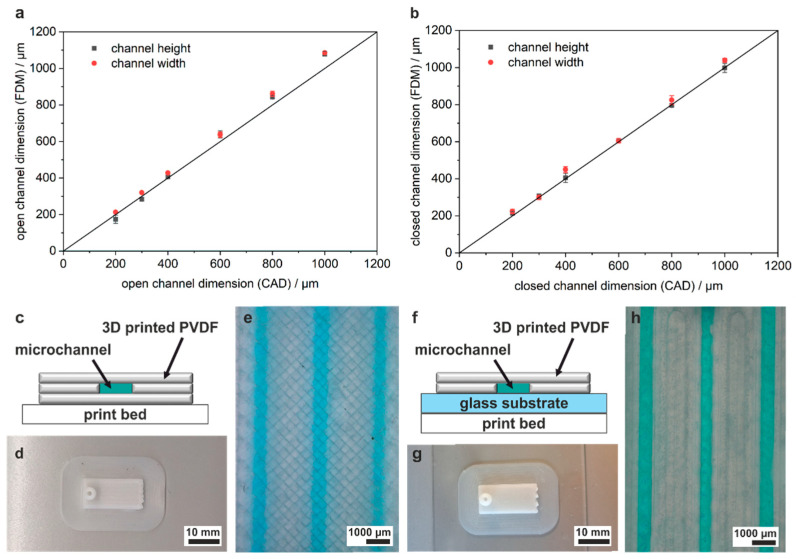
Accordance of FDM-printed microchannels to CAD model and strategy to increase transparency. (**a**) Mean CAD accordance and standard deviation of 3D-printed channel height and channel width compared to the CAD model for open channels, measured at three different positions. All open channels were printed with high accordance to the CAD values. (**b**) CAD accordance of 3D-printed channel height and channel width compared to the CAD model for embedded channel structures. Channels down to 200 µm × 200 µm were successfully printed. An embedded channel width of 100 µm × 100 µm resulted in blocked channels. All other embedded channels were printed with high accordance to the CAD values. (**c**) Schematic of the setup when printing directly on the print bed. The channel bottom is also 3D-printed, which results in a lower optical transmission of the chips. (**d**,**e**) Three-dimensionally printed chip and channels filled with dyed water utilizing the strategy depicted in **c**. The channels are challenging to observe due to the low transparency of PVDF. (**f**) Schematic of the improved printing protocol, which prints the channel without any bottom layer directly onto a glass substrate. Transmission into the channels is only limited by the optical properties of the glass itself. (**g**,**h**) Three-dimensionally printed chip and channels filled with dyed water utilizing the strategy from (**f**). The channels are clearly visible, as only the glass is limiting the transmission of light.

To optimize the dimensional accuracy of FDM-printed microstructures, a calibration structure with open and embedded channels was printed, with variations in the wall infill flowrate. This parameter has a crucial role in determining the dimensions of channels, as it governs the precision with which the material is deposited. The calibration structure featured square-shaped channels, which ranged from a width and height of 1000 µm × 1000 µm down to 100 µm × 100 µm. The CAD structures and the 3D-printed structures can be seen in Figure 1c,d. Optimal dimensional accuracy was achieved with a flowrate for wall structures of 87%. The top view of open and embedded channels is shown in Figure 1e,f. Embedded channels were filled with dyed water to demonstrate functionality. All embedded channels down to a cross-section of 200 µm × 200 µm were successfully filled. To characterize the dimensional accuracy, the 3D-printed width and height of open and embedded channels was plotted against the expected values from the CAD design for the calibration structures. Open and embedded channels were fabricated with high accordance to the CAD values down to 200 µm × 200 µm (Figure 2a,b). The 3D-printed parameters for layer height and line width were compared to the CAD parameters by printing a cube of 1 × 1 × 1 cm^3^. The 3D-printed height of 9736 µm equals to an individual layer height of 97.4 µm, and the cube width of 9751 µm equals to a line width of 341.3 µm, closely matching the CAD values of 100 µm layer height and 350 µm line width.

PVDF is a semi-crystalline polymer with a crystallinity of 35–70%, thus the transparency is lower than other FDM filaments like PLA, PET, or PS [40]. Figure 2e shows channels viewed through the translucent PVDF. In order to increase transmission and improve visualization of the channels, open channels were sealed with a glass slide. This was carried out in a one-step process by printing open channels on top of a glass slide. The setup is shown in Figure 2f. (Figure 2c,d,f,g). In previous studies, we utilized a similar strategy for FDM printing of PMMA and PS [17,18]. Using this setup, the transmission of the chip is only limited by the absorption of the glass substrate. Hence, the visibility of dyed water upon filling the embedded channels is significantly enhanced (Figure 2e,h).

### 3.2. Characterization of FDM-Printed PVDF Chips

**Figure 3:**
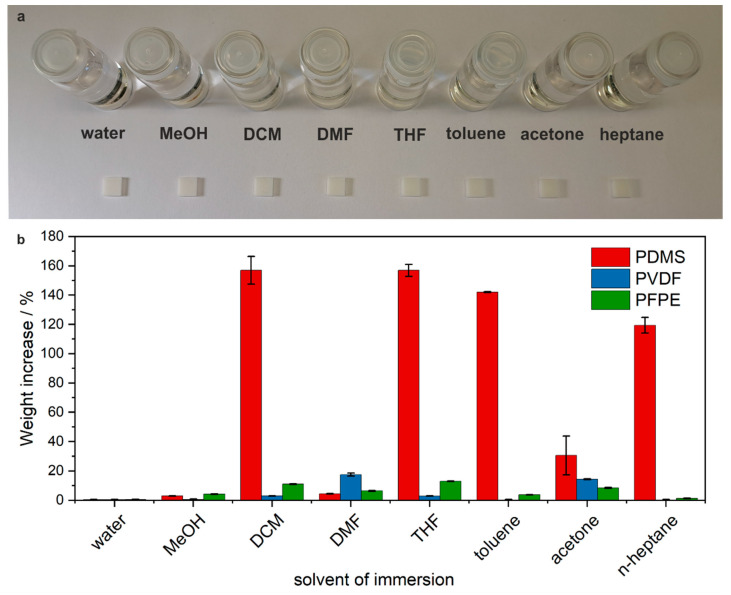
Assessment of chemical resistance of FDM-printed PVDF. (**a**) Blocks of PVDF (10 mm × 10 mm × 3 mm) after 24 h immersion in the respective solvents. Experiments were conducted in sets of three. No structural damage was observed after the swelling. (**b**) Mean weight increase and standard deviation of the immersed PVDF blocks compared to cast and cured PDMS and PFPE methacrylates.

The chemical resistance of FDM-printed PVDF structures was assessed by performing solvent compatibility tests. For this, a total of 24 3D-printed PVDF blocks (10 mm × 10 mm × 3 mm) were immersed in sets of three for 24 h into 20 mL of water, methanol, DCM, DMF, THF, toluene, acetone, and *n*-heptane, respectively. The experiment was repeated with cast PDMS and photo-crosslinked PFPE methacrylates as a benchmark. As seen in Figure 3a, the immersed PVDF structures show no structural damage after 24 h of immersion. The weight difference before and after swelling was calculated for all three materials and is shown in Figure 3b. As seen, the PVDF blocks show very low swelling compared to PDMS and comparable low swelling to the blocks made from PFPE methacrylates, with a maximum swelling in DMF of 17.6 wt%.

Furthermore, the roughness of the 3D-printed structures was analyzed by WLI and Gwyddion in three positions on an area of 1.6 × 1.6 mm^2^ (Figure S1, Supporting Information). The values calculated in Gwyddion for the surface roughness S_q_ were 3.85 µm, 3.85 µm, and 3.95 µm, and the values for the mean line roughness R_a_ were 0.146 µm, 0.175 µm, and 0.148 µm. The porosity of 3D-printed PVDF was also investigated by scanning electron microscopy. Analysis of the bulk phase and surface showed no pores within the 3D-printed structures (Figure S2, Supporting Information).

### 3.3. Three-Dimensional Printing of Functional PVDF Chips

**Figure 4:**
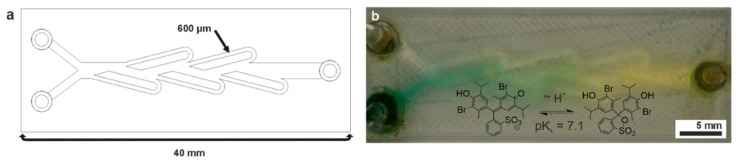
FDM fabrication of a 3D-printed Tesla mixer. (**a**) Schematic of the Tesla mixer design. The minimum outer channel width and height is 600 µm. (**b**) Three-dimensionally printed Tesla mixer and reaction scheme. The chip was filled with 10 mM p-toluenesulfonic acid in DMF mixed with 0.1 mM bromothymol blue and 1 mM triethylamine in DMF. Moreover, 1:3 (vol/vol) water is added to the latter solution in order to express the blue color of bromothymol blue.

Microchips for common microfluidic applications like fluid mixing were fabricated to demonstrate the functionality of 3D-printed PVDF microfluidics. The CAD design of a microchip featuring a Tesla mixer unit is shown in Figure 4a. The minimum channel dimensions were 600 µm height and width, with a total length of the chip of 40 mm. The chips were filled by attaching PTFE tubes to the 3D-printed chip-to-world interface, which was connected to a syringe pump. The connection of the tubes to the chips was additionally supported by epoxy glue. Flowrates up to 10 mL/min were tested, and no leakage was observed. Due to the chemical resistance of the PVDF, the printed chips can be used for applications that common FDM materials would not endure. As a showcase of the chemical resistance, the Tesla mixer was used to protonate bromothymol blue in DMF. For this reaction, one inlet was filled with DMF containing 10 mM *p*-toluenesulfonic acid. The other inlet was filled with 0.1 mM bromothymol blue and 1 mM triethylamine in DMF and water. Bromothymol blue in pure DMF expresses a green color at pH > 7.1. Therefore, 1:3 (vol/vol) water was added, which changed the color of the solution from green to blue. Upon successful mixing, the color changed from blue to yellow due to the protonation of the dye bromothymol blue (Figure 4b). The chip showed no structural damage upon perfusion, demonstrating that PVDF microchips are chemically resistant to being used with aggressive solvents like DMF.

**Figure 5:**
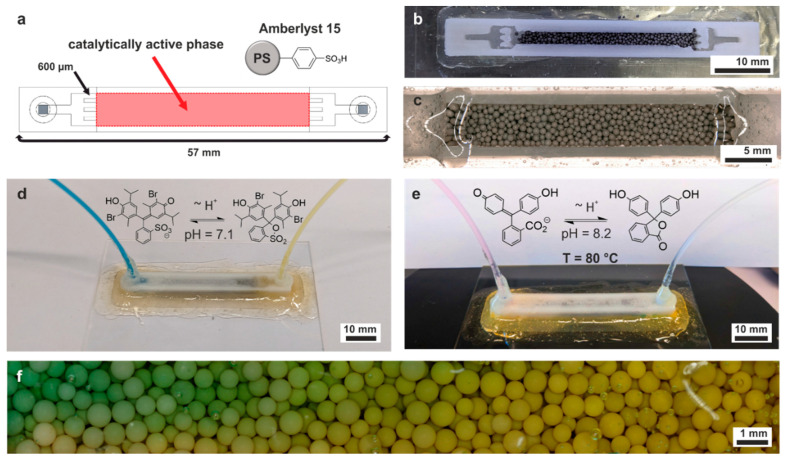
(**a**) Schematic of the 3D-printed microreactor for heterogeneous catalysis. The active phase is added while printing in the center of the chip and held in place with a sieve structure at the front and end of the perfusion channel. For this work, the ion exchange resin Amberlyst 15 was chosen as an acidic active phase. (**b**) Addition of catalytically active material to the microreactor. A print–pause–print protocol is used to fill the flow chamber before the roof is printed. The main channel width appears narrower from the top view due to sloping sidewalls towards the top for easier roof layer fabrication. (**c**) Fabrication of a microreactor with a transparent window into the reaction chamber. A hot glass slide is pressed onto the open channel structure and fixed with epoxy glue. (**d**) Perfusion of the 3D-printed microreactor with the same dyed DMF/water mixture from Figure 4b. No additional acid is mandatory to induce the color change, as the acidic active phase is reacting with the dye. (**e**) Perfusion of the 3D-printed microreactor with water at 80 °C. The chip is placed on a hot plate and perfused with a solution of 0.1 mM phenolphthalein and 1 mM triethylamine in water, which turns from pink to colorless upon protonation. (**f**) Repetition of the perfusion from (**d**) with the reactor featuring a transparent roof from (**c**). Due the highly transparent cover, the color change can be observed in situ.

An advantage of FDM when 3D printing microreactors for reaction-on-chip applications is the possibility of incorporating a catalytically active phase within the chip by utilizing a print–pause–print protocol. Before fabrication of the roof layers, the print is paused and the chamber is filled with any heterogeneous catalyst. The particles are fixed within the perfusion chamber with a 3D-printed sieve structure, which consisted of several pillars with a width of 800 µm and a gap width of 600 µm in between. Amberlyst 15 was chosen as a heterogeneous catalyst, which is a commercial ion exchange resin, composing sulfone groups grafted onto crosslinked PS (Figure 5a). Although being sold mainly for ion exchange operations, Amberlyst 15 has seen widespread usage as a heterogeneous catalyst for acid-catalyzed reactions. For instance, it has been shown to catalyze transesterification [41], Michael addition [42], or Friedel–Crafts reactions [43]. Nevertheless, these examples utilize Amberlyst 15 suspended inside a stirred batch reaction, whereas it has not been used in a continuous 3D-printed microreactor, which effectively overcomes the mandatory catalyst separation step. A linear flow cell was designed with a perfusion chamber with a length of 30 mm, a width of 5 mm, and a height of 4 mm (Figure 5a). While fabrication, the print is stopped twice. The first time, the brim of the structure is fixed with epoxy glue to a glass plate. This ensures that the 3D-printed microreactor can be heated on a hot plate due to full contact with the planar ground and does not require a water heat bath. Due to the high temperature of the print bed, the polymerization of the epoxy glue is finished before the print is complete, preventing any structural change after printing. Prior to printing the roof layers, the print is stopped a second time, and the catalytically active phase is added to the perfusion chamber (Figure 5b). As Amberlyst 15 swells in solvents, the swelling factor of the solvent of choice has to be calculated prior to filling the chamber in order to fill out the whole perfusion chamber in the swollen state. After fabrication, the microreactor was perfused with distilled water, and the pH value at the reactor outlet was measured with a pH test strip. A neutral pH value was observed, which indicates no leakage of the active phase. Therefore, this reactor can be utilized to conduct any acid-mediated reaction (similar to the chip in Figure 4b). As stated, PVDF is not transparent due to its semi-crystalline structure. As this restricts optical analysis within the reaction chamber, we also designed a microreactor with an open channel structure. The design with 3D-printed roof featured sloped wall structures to aid printing of the roof, whereas no slope is required when sealing the channel with a glass slide. After the print is finished, a hot glass plate is pressed on top of the open perfusion chamber and sealed with epoxy glue (Figure 5c). This way, optical analysis within the perfusion chamber is only limited by the optical properties of the glass slide. The 3D-printed microreactor was filled with the same 3:1 (vol/vol) DMF/water mixture from Figure 5d to demonstrate the color change in bromothymol blue upon protonation.

However, it does not require separation of the acid afterwards, which is needed for the chip in Figure 4b. Furthermore, chips printed from PVDF have higher thermal resistance compared to common FDM filaments. While a majority of them are compatible with water, many of them, like ABS, PS, and PLA, are not compatible with water close to its boiling point, which lies close to or above their Vicat softening point [31]. As a showcase of the superior thermal resistance of the PVDF material, 3D-printed chips were placed on a hotplate set to 90 °C. The temperature of the chip surface was measured with an IR thermometer to be around 80 °C. The chip was filled with a solution of 0.1 mM phenolphthalein and 1 mM triethylamine in water. Upon protonation, the color of the solution turned from pink to white due to the structural changes in the π-system of phenolphthalein (Figure 5e). The chip showed no structural damage upon perfusion, demonstrating that PVDF microchips can be conveniently operated at temperatures of 80 °C. Furthermore, the protonation of bromothymol blue in DMF is repeated with the glass roof reactor from Figure 5c. A gradient within the perfusion chamber from blue to yellow can be observed (Figure 5f).

**Figure 6:**
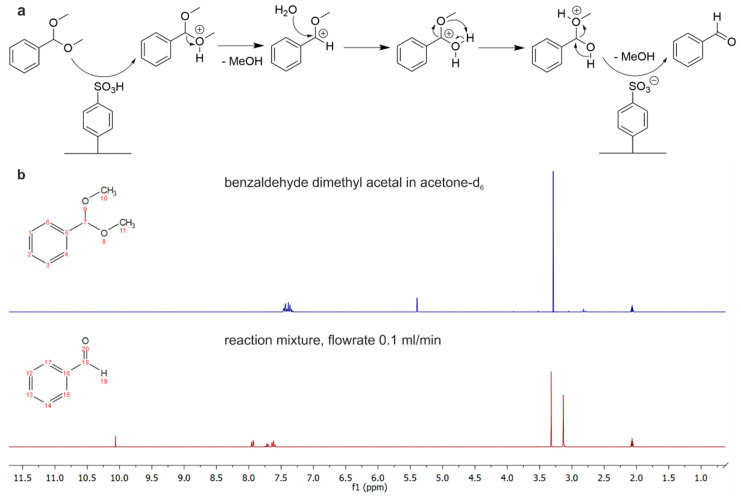
Heterogeneously catalyzed deprotection of an acetal performed on the 3D-printed PVDF microreactor. (**a**) Mechanism of the reaction catalyzed by Amberlyst 15. The reaction was run in acetone-d_6_ with 1.5 vol% water added, and the mixture of the outlet was collected inside an NMR tube. (**b**) ^1^H-NMR in acetone-d_6_ of the starting material benzaldehyde dimethyl acetal (blue) and the reaction mixture (red) retrieved from the microreactor, which was perfused at a flowrate of 0.1 mL/min. The reaction can be monitored via the disappearance of the starting material proton signal at position 7 in the uncluttered σ = 5.40 ppm. No methine acetal proton can be observed, indicating a conversion of more than 99%.

As a demonstration of a heterogeneously catalyzed reaction, the chip was used for an acid-catalyzed deprotection of acetals to aldehydes. This reaction plays a vital role in orthogonal carbonyl chemistry, as aldehydes have a high reactivity and therefore often require protection during a series of chemical transformations of the respective molecule [44,45]. Usually, the reaction is performed under homogeneous conditions, which require an additional separation step to isolate the product [46]. However, if the reaction is performed on-chip, the product can be conveniently collected at the reactor outlet in a one-step process. The chip was filled with a solution of 2 mM benzaldehyde dimethyl acetal in acetone and 0.15 vol% water at a flowrate of 0.1 mL/min at 20 °C. The dimethyl protection group is catalytically cleaved in acidic media within the chip (Figure 6a). The reaction was run in acetone-d_6_ to allow direct ^1^H-NMR measurement of the reaction mixture (Figure 6b). Analysis was performed on a Bruker Avance III HD 300 MHz NMR spectrometer. The reaction is monitored by the disappearance of the methine acetal proton at position 7 at f1 = 5.40 ppm. Alternatively, the reaction can be monitored by the evolution of the aldehyde proton signal at position 19 at 10.06 ppm. The starting material proton signal disappeared completely, indicating a conversion of more than 99%. This demonstrates that the 3D-printed microreactors can be conveniently used for common reactions in organic chemistry that conventional FDM thermoplasts would restrict.

## 4. Conclusions

In this study, we demonstrated the fabrication of chemically and thermally microfluidics by FDM printing of PVDF. Functional microfluidic channels down to 200 µm channel widths and heights were successfully 3D-printed. Furthermore, a protocol was developed to enhance the optical properties of embedded microchannels by printing directly on a glass substrate. The chemical resistance of 3D-printed structures was assessed by swelling in various solvents overnight. PVDF structures showed superior chemical resistance compared to PDMS and comparable chemical resistance to crosslinked PFPE methacrylates. Functional chips for common microfluidic applications like fluid mixing were fabricated and operated with DMF without any structural damage. Furthermore, we developed a facile print–pause–print protocol for 3D-printed microreactors that can be filled with a heterogeneous catalyst within the perfusion chamber. Applications that require optical analysis can also be performed on microchips sealed with a transparent glass slide as the roof structure of the perfusion chamber. We demonstrate the functionality of these chips by performing acid-catalyzed acetal deprotection in acetone with a conversion of more than 99%.

Utilizing our 3D printing protocols, it is possible to fabricate microfluidic chips for applications that require higher chemical resistance than most commercially available filament types. Therefore, our work enables facile chip generation for applications like on-chip chemical synthesis that previously required laborious and time-consuming manufacturing methods like the structuring of glass.

## Data Availability

The original contributions presented in this study are included in the article/Supplementary Materials. Further inquiries can be directed to the corresponding authors.

## Supplementary Materials

The following supporting information can be downloaded at: https://www.mdpi.com/article/10.3390/mi15111391/s1, Figure S1: Roughness measurements of 3D printed PVDF. (a–c) The WLI measurement was conducted at three positions and the roughness was analyzed with Gwyddion to be (a) R_a_ = 0.146 µm and S_q_ = 3.85 µm, (b) R_a_ = 0.175 µm and S_q_ = 3.85 µm and (c) R_a_ = 0.148 µm and S_q_ = 3.95 µm. Figure S2: Scanning electron microscopy of 3D printed PVDF. (a–c) SEM pictures of the top surface of 3D printed PVDF. (d) Sideview on a cut of 3D printed PVDF. No air inclusions or micropores can be seen within the material.
