# Supplementary material for: Fused Deposition Modeling of Chemically Resistant Microfluidic Chips in Polyvinylidene Fluoride
Source: Micromachines (Basel). 2024 Nov 17;15(11):1391. doi: 10.3390/mi15111391 (PMC11596735; doi:10.3390/mi15111391)
Supplement: Supplementary file 1 [file micromachines-15-01391-s001.zip › micromachines-3266509-supplementary.pdf]

Supporting Information

Fused Deposition Modeling of Chemically-Resistant Microfluidic Chips in Polyvinylidene Fluoride

Christof Rein, Leonhard Hambitzer, Zahra Soraya, Han Zhang, Henning J. Jessen, Frederik Kotz-Helmer, and Bastian E. Rapp

The surface roughness R_a and S_q were measured and calculated using WLI and Gwyddion at three positions on an area of 1.6 × 1.6 mm². The three WLI measurements can be seen in Fig. S1. The values calculated in Gwyddion of the surface roughness S_q were 3.85 µm, 3.85 µm and 3.95 µm, which is expected due to the laminate structure of the FDM printed parts. When measured parallel to the writing direction between the laminates, the values of the mean line roughness R_a were 0.146 µm, 0.175 µm and 0.148 µm.

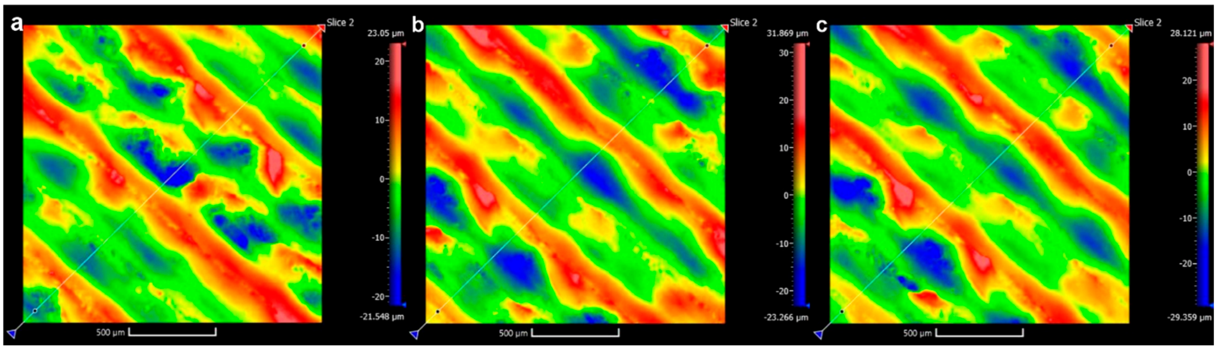

Figure S1: Roughness measurements of 3D printed PVDF. (a-c) The WLI measurement was conducted at three positions and the roughness was analyzed with Gwyddion to be (a) $R_a = 0.146$ µm and $S_q = 3.85$ µm, (b) $R_a = 0.175$ µm and $S_q = 3.85$ µm and (c) $R_a = 0.148$ µm and $S_q = 3.95$ µm.

Scanning electron microscopy was used to analyze the porosity of the 3D printed PVDF. SEM images were taken from the top (Fig. S2a-c) and side view after cutting a sample (Fig. S2d). Surface and bulk images show neither air inclusions, nor micropores in the 3D printed material.

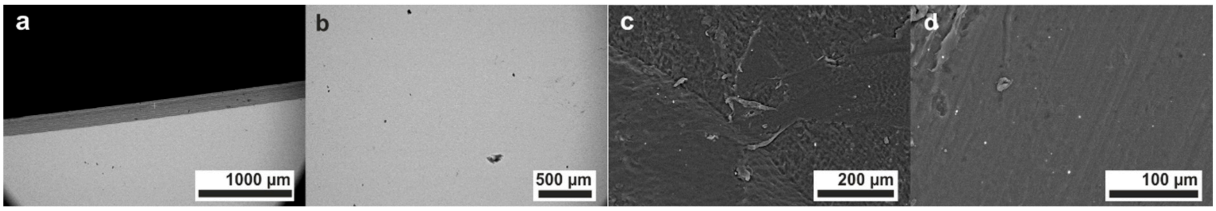

Figure S2: Scanning electron microscopy of 3D printed PVDF. (a-c) SEM pictures of the top surface of 3D printed PVDF. (d) Sideview on a cut of 3D printed PVDF. No air inclusions or micropores can be seen within the material.
